# PW01-024 – Phenotypic analysis of a MEFV negative FMF cohort

**DOI:** 10.1186/1546-0096-11-S1-A77

**Published:** 2013-11-08

**Authors:** A Soriano, D Rigante, L Cerrito, C Fonnesu, L Sicignano, A Gallegos, R Manna

**Affiliations:** 1 Periodic Fevers Research Centre, Catholic University of Sacred Heart, Rome, Italy; 2 Department of Pediatrics, Catholic University of Sacred Heart, Rome, Italy; 3 Nuestra Senora del Prado Hospital, Talavera de la Reina, Spain

## Introduction

Familial Mediterranean fever (FMF) is an inherited autosomal recessive disorder, ethnically restricted and commonly found among individuals of Mediterranean descent, caused by MEditerranean FeVer gene (*MEFV*) mutations on the chromosome 16. It is the most frequent periodic febrile syndrome among autoinflammatory syndromes. Eighty % of patients with FMF have *MEFV* mutations, while around 20% do not have mutations.

## Objectives

We analysed epidemiological and clinical characteristics, as well as treatment schedules of a large cohort of FMF patients without any *MEFV* mutations, who responded to colchicine, in order to identify further clinical features of this specific subgroup.

## Methods

Epidemiological and clinical details of 344 patients attending the Periodic Fevers Research Centre in a period of 15 years were analysed. We selected patients without *MEFV* mutations, in whom diagnosis was established by the Tel-Hashomer criteria. We finally compared the clinical findings of *MEFV*-negative population with the *MEFV*-positive one.

## Results

Genetic testing by *MEFV* analysis was performed in all patients (n = 344); 41 patients (14%, 20 males and 21 females) negative for *MEFV* mutations were selected and studied. Similarly with *MEFV* positive patients, in our case-series, most *MEFV-*negative ones came from Southern and Central Italy. The mean age of FMF onset was 21.8 years, differently from what observed in *MEFV-*positive population, in which the mean age was 15. The frequency of attacks went from less than 1 attack/month (in 26%) to 1-2 attacks/month (in 54%) and more than 2 attacks/months (in 19.5%). The mean duration of each attack was 83.9 ± 8.91 hours. The typical clinical signs of FMF attacks were: fever (T max 39.4°C±0.12, present in 100% of patients), articular pain (76%), abdominal pain (63.4%), oral aphthosis (44%), and chest pain (37%). Thirty-one out of 41 patients had joint involvement in terms of arthritis (21.5%), arthralgias (25%), arthromyalgias (32%), and myalgias (21.5%). Attacks were controlled with a mean dose of colchicine of 1.5 mg/day in all patients (vs a mean dose of 1.3 mg/die in the *MEFV-*positive population). No statistically significant difference was detected in terms of frequency and duration of attacks, as well as in symptoms distribution and colchicine dosage between *MEFV-*negative and positive populations.

## Conclusion

Analysis of our MEFV-negative series of Italian patients revealed a higher prevalence of late-onset FMF, whereas the percentage distribution of symptoms was similar to *MEFV*-positive patients. These results support the hypothesis of involvement of other low-penetrance genetic systems in the FMF clinical expression.

## Disclosure of interest

None declared
